# The clubfoot assessment protocol (CAP); description and reliability of a structured multi-level instrument for follow-up

**DOI:** 10.1186/1471-2474-6-40

**Published:** 2005-07-18

**Authors:** Hanneke Andriesse, Gunnar Hägglund, Gun-Britt Jarnlo

**Affiliations:** 1 Departments of Orthopedics, Lund University Hospital, SE-221 85 Lund, Sweden; 2 Departments of Health Science, Division of Physical Therapy, Lund University, Lasarettsgatan 7, SE-221 85, Sweden

## Abstract

**Background:**

In most clubfoot studies, the outcome instruments used are designed to evaluate classification or long-term cross-sectional results. Variables deal mainly with factors on body function/structure level. Wide scorings intervals and total sum scores increase the risk that important changes and information are not detected. Studies of the reliability, validity and responsiveness of these instruments are sparse. The lack of an instrument for longitudinal follow-up led the investigators to develop the Clubfoot Assessment Protocol (CAP).

The aim of this article is to introduce and describe the CAP and evaluate the items inter- and intra reliability in relation to patient age.

**Methods:**

The CAP was created from 22 items divided between body function/structure (three subgroups) and activity (one subgroup) levels according to the International Classification of Function, Disability and Health (ICF). The focus is on item and subgroup development.

Two experienced examiners assessed 69 clubfeet in 48 children who had a median age of 2.1 years (range, 0 to 6.7 years). Both treated and untreated feet with different grades of severity were included. Three age groups were constructed for studying the influence of age on reliability. The intra- rater study included 32 feet in 20 children who had a median age of 2.5 years (range, 4 months to 6.8 years).

The Unweighted Kappa statistics, percentage observer agreement, and amount of categories defined how reliability was to be interpreted.

**Results:**

The inter-rater reliability was assessed as moderate to good for all but one item. Eighteen items had kappa values > 0.40. Three items varied from 0.35 to 0.38. The mean percentage observed agreement was 82% (range, 62 to 95%). Different age groups showed sufficient agreement. Intra- rater; all items had kappa values > 0.40 [range, 0.54 to 1.00] and a mean percentage agreement of 89.5%. Categories varied from 3 to 5.

**Conclusion:**

The CAP contains more detailed information than previous protocols. It is a multi-dimensional observer administered standardized measurement instrument with the focus on item and subgroup level. It can be used with sufficient reliability, independent of age, during the first seven years of childhood by examiners with good clinical experience.

A few items showed low reliability, partly dependent on the child's age and /or varying professional backgrounds between the examiners. These items should be interpreted with caution, until further studies have confirmed the validity and sensitivity of the instrument.

## Background

Clubfoot is a collection of different abnormalities [1-5] with different etiologies [3,6]. Consequently, severity varies with difficulties in evaluating treatment strategies and outcome results.

Most assessment instruments available for clubfoot aim towards classification or cross-sectional outcome and concentrate on variables belonging to the domains of body functions and structures [6-13]. Variables on activity and participation are sparsely used and addressed only generally [8,12-16]. Teasing, for example, is addressed in one patient based questionnaire [17]. A literature review on Medline, Libris and Elin shows that reliability and validation studies are rare [13,17-19] and regard only six out of a numerous amount of instruments described in clubfoot articles [6,15].

The International Classification of Function, Disability and Health (ICF), developed by the World Health Organization (WHO), is a classification of health and health related domains that describe body function and body structure, activity and participation [20,21]. For studies on outcome, the ICF can be used as a tool to systematically describe measures according to these domains.

The lack of an instrument that is useful during the child's growth, and follows the guidelines of the ICF, led to the development of the Clubfoot Assessment Protocol (CAP). The aims of this study were to *i*) describe this new instrument, *ii*) to investigate item inter-rater reliability between two experienced clinicians with different professional backgrounds, *iii*) to investigate item intra-rater reliability and *iv*) to investigate the influence of age on reliability.

## Methods

### The Clubfoot Assessment Protocol (CAP)

The purpose of the CAP is to provide an overall profile of the clubfoot child's functional status within the domains of body function/structure and activity on single assessment occasions and over time. Furthermore, the CAP aims to provide structure and standardization for follow-up procedures from 0 to11 years of age in daily clinical decision making. It is an observer administered test. The selection of important items to be included in the protocol and scoring system was an act of balance between considerations of clinical utility and scientific interest. Literature studies, expert opinions and clinical experience on what patients /parents present as important factors formed the platform for the CAP prototype.

The CAP (shown in its entirety, as used in daily practice on side 19), (Table 3) contains 22 items in four sub-groups: mobility (8 items), muscle function (3 items), morphology (4 items), and motion quality I and II (7 items). The first three sub-groups relate to body function/structures and the last to activity according to ICF-2001 [8]. Questions about pain, stiffness and daily activity /sport participation are standard. These subjective items are not included in this reliability study.

Each item is described in a manual along with the criteria for scoring. The scoring is divided systematically in proportion to what is regarded as normal variation and its supposed impact on perceived physical function ranging from 0 (severe reduction/ no capacity) to 4 (normal). Score grading can vary between 3 to 5 levels. For sub-groups the sum of the items scores are calculated and can be visualized as profiles (transformed to a 0–100 scale score, with 0 = extremely deviant and 100 within normal variance; sub-group transformation score = actual score/maximal possible score × 100). Missing item assessment is treated by submitting the average scoring for that item. The CAP is not intended for total scores.

Administration time varies between 10–15 minutes dependent on the child's cooperation. Seven items assess motion quality and are age dependent. At the age of three years all children are presumed to be able to perform Motion Quality part I. At the age of 4 all children are also expected to be able to perform Motion Quality part II. Knowledge and experience on normal child neuro-motor development is a prerequisite for enabling proper assessment of the sub-groups muscle function and movement quality.

### Procedure

The reliability study took place over a four month period at routine follow-ups at the clubfoot unit and in a normal clinical setting. The project was regarded as quality control in clinical work. The children were familiar with the examiners. Parents and older children were informed about the testing procedure of the instrument and its importance in increasing the quality of our follow-up program. They were also informed that they could withdraw whenever they wanted. They all gave their consent to participate.

Two examiners, one physical therapist (HA) and one pediatric orthopedic surgeon (GH), both well acquainted with clubfoot problems, assessed consecutively and independently of each other the children in random order. Both had been participants in developing the protocol. HA had clinical experience working with the CAP. GH carefully studied the manual and the protocol before entry. After the first eight patients, the two observers consulted with each other before continuing. To enhance the stability of the phenomenon tested and to prevent the children of getting bored and tired, the examiners took turns in instructing the children while testing the items of domain "motion quality".

The intra-rater reliability test was done by HA.

### Patients

In the inter- rater study, 13 girls and 35 boys born with idiopathic clubfoot, median age of 2.1 years (range, 0 to 6.7 years) were assessed. Twenty-seven children had unilateral and twenty-one had bilateral clubfoot, which gave a total of 69 assessed feet. The feet's severity spectrum in new-born ranged from very mild to very severe [10]. The feet were assessed in different phases of our treatment program. This includes intensive stretching and manipulations on a daily basis during the first 2 month after birth supplemented with an adjustable splint worn 22 hours a day. At the age of 2 month in most cases an Achilles tenotomy and posterior-medial release was needed followed by a 5 week period of casting. At the age of 4.5 month old these children's clubfeet were fully corrected and treatment continued with a special designed dynamic orthosis. In the beginning these orthosis were used 18 hours a day and later on only at night (minimum of 8 hours) until four years of age.

The children were divided into three age groups:

I. Newborn – walking debut (n = 22 feet, median age 3.2 months, range 0 to 1.1 years).

II. Walking debut – four years (n = 25 feet, median age 2.1 years, range 1.2 to 3.9 years).

III. Four years – seven years (n = 22 feet, median age 4.9 years, range 4.0 to 6.7 years).

The intra – rater portion of this study consisted of 20 children, considered to be in a clinical stable phase and a median age of 2.5 years (range, 4 months to 6.8 years). A total of 32 feet, were assessed dispersed in the three age groups as following; 8:14:10. The mean re-examination time was 2.1 months (range, 0.5 to 3.0 months).

Most missing values were seen in age group II in the sub-group motion quality, especially for heel and toe walking (12 out of 25 assessments). This was caused by immaturity in the motor development. In three cases, the child refused to co-operate with one or the other of the observers (Table 2).

**Table 2:** Inter-rater reliability. Unweighted Kappa values, confidence interval (CI) and overall agreement (Po)in percentage for the three age groups separately.

		Age group I			Age group II			Age group III		
Item	S	n	Kappa (95% CI)	Po %	N	Kappa (95%CI)	Po%	N	Kappa(95%CI)	Po %

1. Dorsiflexion	5	22	0.94 (0.81–1.00)	95	25	0.44 (0.16–0.71)	68	22	0.73 (0.5–0.96)	82
2. Plantarflexion	5	22	0.65 (0.34–0.96)	82	25	0.43 (0.14–0.71)	64	20	0.77 (0.35–1.00)	95
3. Subtalar	5	22	0.74 (0.54–0.95)	82	23	0.26 (-0.17–0.69)	68	22	0.71 (0.46–0.96)	82
4. Derotation	5	22	0.53 (0.30–0.76)	73	25	0.65 (0.02–1.00)	96	22	0.62 (0.32–0.93)	82
5. Adduktion	5	22	1.00 (0.00–0.00)	100	25	0.58 (0.23–0.92)	84	22	0.53 (0.21–0.84)	73
6. Tightness	4	22	0.66 (0.43–0.90)	77	23	0.41 (0.10–0.73)	84	21	0.85 (0.65–1.00)	90
7. Flx.dig.long	3	13	0.00 (0.00–0.00)	77	25	-0.06(-0.14–0.0)!	88	18	0.00 (0.00–0.00) !	94
8. Flx.dig.hall.	3	13	0.00 (0.00–0.00)	85	25	0.83 (0.52–1.00)	96	18	0.00 (0.00–0.00) !	94
9. M. Peroneus	3	22	0.58 (0.30–0.86)	73	25	0.54 (0.24–0.85)	76	22	0.57 (0.26–0.88)	72
10. M. ext.dig.ln	3	22	0.21 (0.02–0.40)	64	24	0.60 (0.21–0.99)	84	22	0.26 (-0.15–0.66)	72
11. M. sol/gastr.	3	20	0.52 (0.12–0.93)	80	21	0.00 (0.00–0.00) !	81	22	0.00 (0.00–0.00)!	91
12. Tib.rotation	3	22	0.74 (0.40–1.00)	91	25	0.71 (0.41–1.00)	88	22	0.62 (0.16–1.00)!	91
13. Calc.pos.	3	22	0.78 (0.56–1.00)	86	21	-0.07(-0.2–0.04)!	84	22	0.43 (0.00–0.98)	86
14. Forefootpos.	3	22	0.82 (0.60–1.00)	82	21	0.63 (0.26–1.00)	86	19	0.41 (0.06–0.75)	68
15. Foot arch	3	22	1.00	100	21	0.00 (0.00–0.00)	95	21	0.43 (0.10–0.75)	81
16. Walking	4		n.a		24	0.71 (0.45–0.97)	83	22	0.64 (0.37–0.92)	77
17. Toe walking	4		n.a		12	0.60 (0.15–1.00)	83	22	0.38 (0.02–0.74)	68
18. Heelwalking	4		n.a		12	0.37 (0.06–0.68)	58	22	0.52 (0.23–0.81)	68
19. Squatting	4		n.a		18	0.50 (0.08–0.92)	78		n.a	
20. Running	4		n.a		18	0.67 (0.35–0.98)	83	22	0.13 (-0.21–0.47)	45
21. One legstand	4		n.a			n.a		22	0.66 (0.41–0.91)	77
22. Hop 1 leg	4		n.a			n.a		22	0.94 (0.82–1.00)	95

S = amount of scales, n = number of feet assessed

! = limited variation of cell frequency

n.a. = not applicable, due to age dependent activities.

The distribution of the assessments scores were more equally spread in the age group I and for all ages together. Age group II and III had assessment shifting more to the right of the scale for the first 15 items.

### Statistics

Unweighted Kappa (*k*) statistics for agreement were used [22-24] with 95% confidence interval. It calculates agreement beyond chance. As kappa values can become unstable under certain conditions [24,25], the observed percentage agreement (P*o*) was also calculated. A P*o > *75% was regarded as good. In cases with limited distribution of cell frequency, the P*o *was preferred instead of *k*. The amount of categories is also regarded as kappa values decrease when categories increase [25]. The kappa has a maximum of 1 when agreement is perfect, but a value of 0 indicates no agreement better than chance, and negative values show worse than chance agreement. According to Altman [22] the kappa values are to be interpreted as follows: <0.20 as poor agreement, 0.21–0.40 as fair, 0.41–0.60 as moderate, 0.61–0.80 as good and > 0.80 as very good agreement.

A good reliability was considered when the kappa value was high, or a low kappa value combined with a high in P*o*. A sufficient reliability was considered in cases with fair- moderate kappa values and good percentage agreement.

The SPSS 12.00 and StatXact (version 3) was used for the statistical analyses.

## Results

### Inter- rater reliability and age influence

Altogether 1196 assessments were made by each examiner.

For all children (n = 48, 69 clubfeet), 18 out of 22 items had kappa values > 0.40 (range, 0.52 to 1.00). Two items ranged from 0.35 to 0.36 but had good Po (Table 1). Item 7 had a negative kappa score caused by skewed frequency distribution but a good Po = 87%. Item 20 had a kappa value of 0.38 and Po = 62% and is assessed as fair agreement.

**Table 1:** Inter- and intra reliability. Unweighted Kappa values, confidence interval (CI) and overall agreement (Po) for the inter- and intra rater reliability tests and age interval 0 – 7 years.

		Inter-rater			Intra-rater		
Item	S	n	Kappa (95% CI))	Po (%)	n	Kappa (95% CI)	Po (%)
1. Dorsiflexion	5	69	0.73 (0.60–0.86)	85	32	0.72 (0.50–0.94)	84
2. Plantarflexion	5	67	0.62 (0.45–0.79)	79	32	0.66 (0.41–0.91)	66
3. Subtalar	5	67	0.65 (0.49–0.79)	79	32	0.83 (0.66–1.00)	91
4. Derotation	5	69	0.62 (0.45–0.79)	84	32	0.93 (0.76–1.10)	97
5. Adduktion	5	69	0.71 (0.54–0.88)	85	32	0.94 (0.83–1.05)	97
6. Tightness	4	66	0.68 (0.53–0.83)	80	32	0.61 (0.35–0.87)	78
7. Flx.dig.long	3	56	-0.03 (-0.09–0.03) !	87	32	0.72 (0.36–1.08)	94
8. Flx.dig.hall.	3	56	0.57 (0.20–0.94) !	93	32	0.54 (0.27–0.81)	81
9. M. Peroneus	3	69	0.60 (0.44–0.76)	75	26	0.63 (0.34–9.92)	94
10. M. ext.dig.lgn	3	68	0.36 (0.16–0.56)	75	28	1.00	100
11. M. sol/gastr.	3	63	0.35 (0.05–0.60)	84	24	1.00	100
12. Tib.rotation	3	69	0.70 (0.50–0.90)	90	31	0.84 (0.63–1.00)	94
13. Calc.pos.	3	65	0.63 (0.43–0.83)	85	32	0.80 (0.53–1.00)	94
14. Forefootpos.	3	62	0.63 (0.44–0.82)	82	32	0.82 (0.63–1.00)	91
15. Foot arch	3	64	0.5 (0.30–0.86) !	92	31	1.00	100
16. Walking	4	46	0.68 (0.50–0.86)	80	27	0.81 (0.63–0.99)	89
17. Toe walking	4	34	0.49 (0.21–0.77)	73	15	0.61 (0.27–0.95)	80
18. Heelwalking	4	34	0.47 (0.23–0.71)	65	15	0.79 (0.52–1.00)	87
19. Squatting	4	19	0.56 (0.18–0.94)	79	19	0.80 (0.54–1.00)	89
20. Running	4	40	0.38 (0.14–0.62)	62	27	0.78 (0.59–0.97)	85
21. One legstand	4	22	0.70 (0.45–0.95)	77	9	0.68 (0.28–1.00)	77
22. Hop 1 leg	4	22	0.94 (0.82–1.06)	95	9	0.70 (0.33–1.07)	77

S = amount of scales, n = number of feet assessed

! = limited variation of cell frequency

The two examiners agreed totally in 82% of the assessments (range, 62 to 95%). (Table 2). A one – category disagreement was seen in 17% of the cases, whereas a two-category disagreement was seen in 1 %. We conclude that all but one item had moderate to good agreement.

For age group I, 12 /15 items had kappa values > 0.40 (range, 0.52 to 1.00) (Table 2).

Items 7 and 8 had poor kappa values (kappa = 0.00) due to skewed frequencies but acceptable observer agreement (77% respectively 85%). Item10 had fair reliability with a kappa of 0.21 and Po of 64%.

For age group II, 14/ 20 items had kappa values > 0.40 (range, 0.41 to 0.83). Items 7, 11, 13 and 15 had poor values caused by limited distribution of cell frequency but good observer agreement (respectively 88%, 81%, 84% and 95%). Items 2 and 18 had kappa values of 0.26 and 0.37 respectively, and a Po of 68% and 58% respectively and are regarded as having fair reliability.

For age group III, 16/21 items had kappa values > 0.40 (range, 0.41 to 0.94). Items 7, 8, and 11 had poor kappa values also due to skewed distribution but very good observer agreement (94%, 94% and 91% respectively). Item 17 had a fair kappa value and a Po= 68 %. Item 20 had both poor kappa values and poor observer agreement (45%). Item 19 (squatting) was not assessed in this age group.

Taking into account the kappa values, the Po and amount of scales, no age group showed clearly poor reliability values for its items except for item 20, running, in age group III.

### Intra – rater reliability

A total of 587 assessments were done twice. All items had kappa values > 0.40 (range, 0.54 to 1.00) (Table 1). Total agreement was reached in 89 %. A one-category disagreement was seen in 10 % and a two-category disagreement in 0.3 %.

## Discussion

The CAP protocol items had moderate to very good inter-rater reliability for all the items in the age group 0–7 years and for most of the items when regarding the specific age groups.

The intra-rater test showed good to excellent reliability and indicates a good standardization of the protocol.

Most items in our protocol had moderate to excellent inter-observer reliability especially concerning sub-groups "passive mobility" and "morphology". This is a positive finding in the light of the fine-grained protocol with up to five different categories and the two observers' different professions and different experience with the protocol.

### Methodological issues

Reliability studies in children are difficult to perform. The risk for errors is high as the children's co-operation and task understanding may vary from day to day and between different examiners. A child-friendly environment and familiarity with the examiners are important factors in enhancing reliability. We also wanted a situation that was comparable with a normal clinical setting where the instrument is intended to be normally used. These are the reasons why the investigation was unblinded and no more than two examiners were involved.

The fact that one of the examiners had extensive practical experience with the instrument while the other had only co-operated with the development of the protocol might have influenced the result.

In clinical practice teamwork often is the norm and therefore we chose two different professions. However Flynn et al. [18] observed in his study that including a physical therapist decreases reliability; agreement should be expected to increase if assessment is kept within the same profession.

The children available for our study represented the clubfoot spectrum [6] and illustrated the clinical development. Gender distribution corresponded well with the 3:1 (male/female) ratio normally described [3].

### Statistics

When working with ordered categorical data as, in the case of our protocol, the right way of analyzing agreement is said to be Kappa [22,24]. We chose the unweighted Kappa as we wanted to know how the exact agreement would be for our finely graded instrument. It is more common though to use the weighted Kappa statistics that take into account the degree of disagreement [22]. These values are usually higher. We recalculated our kappa's to weighted and found that the values increased between 0.01 and 0.20. For example, our kappa value for the item "running" in age group III, changed from 0.13 to 0.46 when using weighted kappa statistics. This indicates that we can increase our reliability by combining categories. Within research, the finely graded protocol should be prioritized. Care should be taken when interpreting kappa statistics as the value of kappa depends upon the proportion of subjects in each category [24,26]. Haas [24] emphasizes that kappa becomes unstable under certain conditions. The problem-limited variation occurs when there is a large proportion of agreement and most of the agreement is limited to only one possible rating choice. We saw this problem for example in item 7. When all children between 0–7 years were included, untreated, treated and relapsing feet were assessed which meant that the whole scoring spectrum was used. Problems with limited distribution therefore became less. The older children generally had scores that lay more to the right on the protocol which caused a certain ceiling effect. Thus the CAP detects differences in severity which confirms part of its construct validity.

Another possibility for assessing reliability would have been to calculate statistical differences between the total sub scores for each observer, as Flynn et al. [18] did in their reliability study comparing the Pirani [13] and Dimeglio scores [11]. Another way might be to use the mean difference and calculate the 95% limits of agreement as Altman describes [22]. This could give us information on how much we can expect every new assessment to differ between new examiners and individuals and its clinical relevance.

### Results

We have described the CAP; an alternative assessment tool for both short-and long-term follow-ups of children treated for clubfoot. Our protocol differs from most others through scoring grades with smaller intervals and incorporates a broader assessment on movement quality. It is also intended to be used longitudinally during the child's growth. The focus is primarily on item level and secondary on subgroup level. With sum scores and categorization/classification, important information can be lost and it should therefore be avoided [26,27]. Research profiles can be made for each item-score or subgroup(s) scores from the CAP at a certain time or over a time interval on group or individual level. In daily clinical work, the CAP is a promising tool in increasing the quality of follow -up procedures and clinical decision making through standardization and gives the possibility of a visual feedback. It also will give us the possibility to analyze factors influencing the clubfoot development.

With outcome studies, a holistic approach is of importance. The CAP should be supplemented with a patient- and parent-based questionnaire with items specifically focusing on symptoms and limitations in daily life, such as the patient- based questionnaire developed by Roye et al [17]. The Laaveg- Ponseti [12] rating system also has a score distribution emphasizing the importance of patient satisfaction and participation. Recently, several outcome measures focusing on the child's physical functioning in her or his environment, such as the Pediatric Outcomes Data Collection Instrument (PODCI) [28] and the Activity Scales for Kids (ASK) [29,30], have been developed. The use of these kinds of outcome instruments in the future will increase our knowledge of factors that are probably of more importance for patient satisfaction than range of motion, strength and radiographic changes. In the future these factors will become more and more important when discussing outcome results [10,31,32].

Face validity (whether a test appears to measure what it is supposed to measure) and content validity (the extent to which the measures represents functions or items of relevance given the purpose and matter of issue) [34] are enhanced through the developmental procedure. This is based on literature studies, discussions, clinical experience and patient information. Through clinical trial the tool was adjusted several times during the years used at the clubfoot-clinic and might be further adjusted.

Reliability for the different age groups is, with respect to the difficulties met in assessing children, within acceptable limits. Items, which demanded maturity, co-operation and task comprehension such as muscle function, are more vulnerable for different assessment results as research conditions can change between the observers. This is clearly seen in the total group for item 10, kappa value of 0.36, and item 11 kappa value 0.35.

Distinguishing differences in running quality is not easy to assess which is expressed in a low kappa value of 0.38 (fair). It is a fast movement and to observe slight variations is difficult. In our study nearly all differences lay between slightly deviant and normal.

Wainwright et al. [21] assessed the reliability of four classification systems from Catterall [9], Dimeglio et al. [11], Harrold and Walker [35] and Ponseti and Smoley [3]. These instruments are only comparable with the CAP mobility domain. Nine children (13 clubfeet) were assessed by four examiners at different stages in the first 6-months of life (= 180 examinations). The results showed kappa values varying between 0.14 and 0.77. It is not reported if the kappa is weighted or unweighted. The kappa values for our CAP-mobility items vary between 0.57 – 0.73 for ages 0–7 years and ages 0-walking debut between 0.32 – 1.00. We consider this to be positive in the light of the fine graded scales in our protocol.

### Future research

Further studies on psychometric aspects are ongoing and are needed before the CAP can be used in a scientifically sound way. Changes in items used and item groupings are therefore expected.

## Conclusion

The CAP contains more detailed information than previous protocols. It is a multidimensional observer-administered measurement instrument with the focus on item and subgroup level. It can be used with sufficient reliability independent of age during the first seven years of childhood by examiners with good clinical experience.

A few items showed low reliability, partly dependent on the child's age and /or varying professional backgrounds between the examiners. These items should be interpreted with caution, until further studies have confirmed the validity and sensitivity of the instrument.

## Competing interests

The author(s) declare that they have no competing interests.

## Authors' contributions

HA and GH designed the study and collected the data. HA analyzed the data and drafted the manuscript. HA and G-BJ interpreted the data. HA, GH and G-BJ revised the manuscript. All three authors read and approved the final manuscript.

**Table 3:** The Clubfoot Assessment Protocol (version 1.0)

Name:			Date of birth:		
Date of assessment:			Assessment number:		
Side:	O Left	O Right			
					
Rating	**0**	**1**	**2**	**3**	**4**
**Passive mobility**					
					
1. Dorsiflexion	< -10°	-10°-< 0°	0°- < +10°	+10°- +20°	>+20°
2. Plantar flexion	0°- < 10°	10°- < 20°	20°-< 30°	30°- 40°	>40°
3. Varus/valgus	>20°var	20°-< 10°var	10° -< 0°var	0°- neutral	>0°vlg
4. Derotation	>20°inv	20°-< 10°inv	10°- < 0°inv	0°- 10°evr	>10°evr
5. Add/abd	>20°add	20°-< 10°add	10°- < 0°add	0°- neutral	>0°abd
					
6. Tightness	+ tight	tight		soft-tight	soft
7. Flx.dig.long.	+ reduced		reduced		normal
8. Flx.dig.hall.	+ reduced		reduced		normal
					
**Muscle function (strength)**					
9. M. peroneus	absent/poor		reduced		normal
10. M. ext.dig.long	absent/poor		reduced		normal
11. M. sol./gastr.	absent/poor		reduced		normal
					
**Morphology**					
12. Tibial rotation	+ inw.		inw.		normal
13. Calcaneus position	>10 varus		>0 varus <10		neutral/vlg
14. Forefoot position	>20° add.		>10 add. <20°		add<10°
15. Foot arch	+ cavus		cavus		normal
					
**Motion quality**					
**I**					
16. Walking	+ deviant	deviant		slightly deviant	normal
17. Toe walking	cannot	deviant		slightly deviant	normal
18. Heel walking	cannot	deviant		slightly deviant	normal
19. Squatting	cannot	deviant		slightly deviant	normal
20. Running	+ deviant	deviant		slightly deviant	normal
**II**					
21. One legstand	cannot	deviant		slightly deviant	normal
22. Hop1leg	cannot	deviant		slightly deviant	normal

Extra notes: Structured questions about pain, stiffness, shoe problems, physical condition, activity level, sports and social participation and patient/parent satisfaction.

+ = pronounced / very, var= varus, vlg= valgus, inv = inversion, evr = eversion, add = adduction, abd = abduction. inw = inward rotation, flx.dig.long. = length of M. flexor digiti longus, flx.dig.hall. = length of M. flexor digiti hallucis

^©^The CAP is copyright to Hanneke Andriesse, 2003.

## Pre-publication history

The pre-publication history for this paper can be accessed here:


